# Supplementary material for: Non-disruptive collagen characterization in clinical histopathology using cross-modality image synthesis
Source: Commun Biol. 2020 Jul 31;3:414. doi: 10.1038/s42003-020-01151-5 (PMC7395097; doi:10.1038/s42003-020-01151-5)
Supplement: Supplementary file 5 — Description of Additional Supplementary Files [file 42003_2020_1151_MOESM5_ESM.pdf]

Description of Additional Supplementary Files

File Name: **Supplementary Data 1**

Description CurveAlign analysis results for real SHG collagen fiber orientation and synthesized SHG collagen fiber orientation.

File Name: **Supplementary Data 2**

Description Collagen fiber orientation and alignment analysis across ROIs using CurveAlign on real SHG images and synthesized SHG images.
